# Exploring the experiences of English-speaking women who have moved to Israel and subsequently used Israeli fertility treatment services: A qualitative study

**DOI:** 10.1371/journal.pone.0309265

**Published:** 2024-08-28

**Authors:** Lucy Davies, Gilles de Wildt, Yael Benyamini, Anoushka Ramkumar, Rachel Adams

**Affiliations:** 1 Institute of Clinical Sciences, College of Medical and Dental Sciences, University of Birmingham, Edgbaston, Birmingham, United Kingdom; 2 Bob Shapell School of Social Work, Tel Aviv University, Tel Aviv, Israel; 3 Health Services Management Centre, University of Birmingham, Edgbaston, Birmingham, United Kingdom; Marie Stopes International, PAKISTAN

## Abstract

**Background:**

Israel’s pronatalist cultures result in a social expectation to have children and drive Israel’s fertility rate of 2.9. Israeli policy reflects this through funding unlimited fertility treatment up to two children. Societal pressure to have children exacerbates challenges of fertility treatment. Furthermore, the lack of financial burden creates a culture of perseverance following treatment failures. Whilst the experiences of Israeli women using fertility treatment have been studied, the experiences of women who migrated to Israel and were therefore raised in a different society have not. This study aimed to address this gap in knowledge.

**Methods:**

A qualitative study using semi-structured interviews to investigate the experiences of 13 English-speaking women who utilised Israeli state funded fertility treatment. Participants were located across Israel and were recruited using purposive sampling through social media. Data was analysed using framework analysis.

**Results:**

Despite not being aimed at specific ethnic or religious groups, all respondents were Jewish. Three themes were identified: *1*. *Systemic factors*: The lack of financial burden was positive, however, participants struggled to navigate the bureaucratic healthcare system, especially when experiencing a language barrier. *2*. *Influence of others*: Encountering a cold bedside manner alongside contending with the expectations of a pronatalist society was challenging. Participants utilised support from other migrants who appreciated the same culture shock. Understanding of healthcare professionals regarding shared religious values further improved treatment experiences. *3*. *Impact of journey*: Participants often withdrew socially and the treatment process implicated upon their lives, jobs and relationships.

**Conclusion:**

Navigating a bureaucratic system and pronatalist society are difficulties associated with fertility treatment in Israel. The lack of financial burden and an understanding of religious and cultural beliefs by healthcare providers improved treatment experience. Better provision of resources in English and further research into supporting women who are navigating Israel’s pronatalist society is required.

## Introduction

Israel is widely recognised as having a pronatalist society [1]. Pronatalist societies are associated with a strong family orientation and an expectation to have children [2]. Motherhood is regarded as an essential aspect of life and voluntary childlessness is rare and, for many, socially unacceptable [3, 4]. This is reflected in the finding that 80% of Israelis believe child-rearing is the “greatest joy of life” [5]. Explanatory factors for the desire to reproduce include the biblical commandment to those following Abrahamic religions to ‘be fruitful and multiply’ (Genesis 1:28), the traumas of the Holocaust, and the aspiration to maintain the Jewish population in Israel [1, 6].

Pronatalist cultures are reflected in Israel’s total fertility rate of 2.9 [7]. This is much higher than the average fertility rate in 2021 of 1.58 within the Organisation for Economic Co-operation and Development (OECD) countries [8]. According to The World Bank data, Israel’s total fertility rate has been stable for at least 30 years [7]. Additionally, a 2021 paper describes the fertility rate in Israel as roughly double the rate observed in other economically similar countries [9]. Data from Israel’s Central Bureau of Statistics 2021 report shows that within Israel Jewish women have the highest fertility rate of 3.13 compared with 3.01 for Muslim women, 1.77 for Christian women and 2.00 for Druze women [10]. All Jewish sub-groups are seeing a rise in fertility rate, whereas rates for Muslim, Christian and Druze women have decreased [9].

According to Israel’s Central Bureau of Statistics, its population in December 2022 was estimated at 9,656,000 residents, 73.6% of whom are Jews, 21.1.% Arabs and 5.3% others, including Druze. Throughout 2022 the Israeli population increased by 2.2%, with 38% of this due to migration balance, and the remainder due to natural growth. Overall, 178,000 infants were born in 2022, 74.8% to Jewish mothers, 23.8% to Arab mothers and 1.4% to mothers of Others [11].

Health policy in Israel reflects the social expectation to have children through provision of state funded fertility treatment [12]. All Israeli residents are entitled to healthcare through a National Health Insurance system with compulsory insurance based on a choice of one out of four competing health plans. Workers pay a set health tax as a percentage of their wage towards funding the healthcare system. Although the insurance is comprehensive, covering most emergency, primary and inpatient care, further co-payments for certain services or medications may be required [13]. However, even the basic insurance entitles women to receive unlimited cycles of state funded fertility treatment, including in vitro fertilisation (IVF), until the birth of up to two live children with their current partner. This policy applies regardless of existing children with other partners, marital status or sexual orientation. Women are eligible to receive IVF up to age 45 years old when using their own gametes or up to age 54 in the case of egg donation [14]. This policy facilitates Israel having the highest rate of infertility treatment cycles and number of fertility clinics per capita worldwide [15, 16].

Whilst the policy of state funded fertility treatment allows individuals who otherwise would not be able to have children to become parents, it is important to note the potential burden imposed by fertility treatment, which is exacerbated within the context of pronatalist societies. Universally infertility has been found to be a ‘major life crisis’ associated with feelings such as guilt and helplessness [4, 17]. The high value placed on motherhood by pronatalist states such as Israel increases the vulnerability of fertility patients to psychological distress [5]. Furthermore, considering voluntary childlessness in Israel is virtually unheard of, this puts additional external pressure on women to conceive [14]. This pressure may be exacerbated for religious women since a religious lifestyle is typically child centred, therefore religious childless women often find socialising within their community difficult [18].

Alongside the emotional and psychological burden of the fertility treatment journey there are also physical challenges including breast tenderness, pain at injection sites and risk of ovarian hyperstimulation syndrome [19, 20]. There have been concerns regarding long-term health implications of fertility treatment, particularly the increased risk of certain cancers. However, a 2017 review into the association between fertility medication and cancer risk was reassuring, finding while infertility is a risk factor for breast, endometrial and ovarian cancer, there is insufficient evidence to suggest an association between fertility medication and cancer risk [21].

The social context in Israel has led to a culture of perseverance with fertility treatment [22]. Although women receive information about the odds of treatment success they are often unrealistically optimistic. While this contributes to their wellbeing it also makes treatment discontinuation difficult [14]. Women in Israel are therefore likely to endure treatment for a prolonged period, with a study finding 86% of respondents would undergo as many IVF cycles as needed [20], with some women using as many as 20 treatment cycles [14].

Current literature has addressed the assisted reproductive technology landscape in Israel. Despite there being a sizeable quantity of literature, it is focused on a population of native Israeli women. Jews are encouraged to migrate to Israel and under the Law of Return all Jews have the right to do so and to receive citizen status immediately [23, 24]. Migrants to Israel are unlikely to have grown up in such a highly pronatalist society. Additionally, their native language is unlikely to be Hebrew and they may not be used to the concept of state funded fertility treatment. Demographic data shows that migrants to Israel from lower fertility countries go on to increase their fertility rate [9]. Considering in 2023 there were approximately 45 thousand migrants who relocated to Israel [25], it is important to address this gap in the literature surrounding the experiences of non-native women undergoing fertility treatment. Therefore, this study aimed to investigate the experiences of English-speaking women who migrated to Israel and subsequently used state funded fertility treatment.

## Methods

### Study design

A qualitative methodology, using semi-structured interviews, was selected as it allowed flexibility for in-depth exploration of ideas raised by participants. The use of a topic guide provided structure for the interviewer [26]. This study conformed to Consolidated Criteria for Reporting Qualitative Research (COREQ) guidelines [27].

### Study setting

The study was set in Israel, with the lead researcher based in Tel Aviv for the duration of the study and participants located across Israel. Interviews were conducted via Zoom as this was deemed most appropriate given the COVID-19 pandemic.

### Population

Participants were native English-speaking women who migrated to Israel after age 18 years and had subsequently used at least one full cycle of largely state funded fertility treatment in Israel. Participants were entitled to benefits according to Israeli National Health Insurance thereby allowing them to receive state funded fertility treatment. Women of any marital status, any treatment outcome and with any number of existing children were included.

### Sampling and recruitment

This study utilised purposive sampling, with supplementary snowball sampling in order to improve feasibility of recruitment. Participants were recruited through an advertisement posted in relevant Facebook groups. They included Israel specific parent support groups and fertility support groups. Interested individuals were emailed the participant information sheet and given the opportunity to ask questions. Following receipt of a signed consent form, including consent to participate and consent for recording of the interview, an interview was arranged.

We planned to conduct up to 20 interviews, with recruitment terminated once data saturation was reached. Information was supplied to 19 potential recruits of whom 13 were interviewed. Reasons for non-participation included not meeting the study criteria and being unable to find a suitable time for interview.

### Data collection

Participants were interviewed once, in English, by the lead researcher. Interviews were recorded. Prior to interview commencement, the participant demographic form was completed verbally. This included details regarding previous fertility treatment usage.

An iterative topic guide (S1 File) was used to structure the interviews. Using open questions allowed the flow of each interview to be participant led and also facilitated in-depth discussion of all ideas raised [28]. Ultimately the interviews lasted a mean length of 68 minutes (range 46–95 minutes). Immediately after each interview reflective notes were made regarding new topics raised, any issues identified with the topic guide and whether data saturation was being approached, and reviewed with the research team (RA, GdW, YB).

Following interviewing 13 women, no new themes were being identified, the team therefore agreed that data saturation had been reached. A larger sample size may have highlighted further findings, however, since no new themes were raised, and with time as limiting factor data, collection was concluded.

### Data analysis

Interviews were transcribed intelligent verbatim. Utilisation of the constant comparison method allowed thorough investigation of themes [29].

The Framework analysis approach was used to guide analysis [30]. The clear steps provided by Ritchie and Spencer’s five step approach guided the lead researcher throughout analysis [30]. NVivo 12 software was used for management of data [31]. The lead researcher became familiar with data during transcription and reading of the transcripts. Coding of three interviews and then developing codes into thematic categories allowed identification of a framework. This framework was then applied to the remaining transcripts. Indexed data was charted and summarised using Excel. Finally, themes were formulated from data through an inductive approach and interpretations were made. Multiple transcripts were coded by a secondary researcher and themes were discussed within the research team. The primary researcher is a British, medical student who has existing experience with fertility treatment in the United States. Reflexivity was maintained throughout with the researcher remaining aware of how her background and opinions may have influenced the study and its findings [32].

### Ethics

Ethical approval for this study was received from the University of Birmingham’s BMedSci Internal Research Ethics Committee (IREC2020) and the Tel Aviv University Institutional Review Board (0003890–2). Data cannot be shared publicly because confidentiality was promised, and the level of detail within the transcripts may make interviewees identifiable despite attempts to anonymise them. Data are available from the corresponding author subject to completion of a data sharing agreement.

### Inclusivity in global research

Additional information regarding the ethical, cultural, and scientific considerations specific to inclusivity in global research is included in the Supporting Information (S1 Checklist)

## Results

13 women were interviewed throughout February 2022. Participants had a mean age of 36 years (range 25–50 years) and prior to commencing fertility treatment had conceived between 0 and 3 children through unassisted conception. At the time of interview all participants had either had a live birth or were pregnant through fertility treatment. All participants were Jewish, despite recruitment efforts not being targeted at one specific religious group. Advertisements were posted in various Israel specific Facebook groups, for example, city specific, parenting support and fertility support groups (no groups with religious affiliations for the target population were found). All women had at least a basic understanding of Hebrew and were from North America or the UK. Participant characteristics are displayed in Table 1.

**Table 1:** Participant characteristics table.

Participant characteristics		*n (*N = 13)	%
**Age (years)**	25–30	3	23.1
	31–35	3	23.1
	36–40	5	38.5
	41–50	2	15.4
**Marital status**	Single	1	7.7
	Married	12	92.3
**Religion**	Jewish	13	100
	Other	0	0
**Religiosity**	Secular	1	7.7
	Traditional	1	7.7
	Religious	10	76.9
	Undecided	1	7.7
**Country of birth or early childhood**	US	6	46.2
	UK	5	38.5
	Canada	2	15.4
**Age moved to Israel (years)**	18–20	3	23.1
	21–25	2	15.4
	26–30	7	53.8
	31–35	1	7.7
**Languages spoken**	English	13	100
	Hebrew	13	100
	Other	5	38.5
**Number of children conceived without fertility treatment**	0	7	53.8
	1	2	15.4
	2	3	23.1
	3	1	7.7
**Number of children conceived with fertility treatment**	0	0	0
	1	5	38.5
	2	7	53.8
	3	1	7.7
**Age of fertility treatment commencement (years)**	20–25	4	30.8
	26–30	5	38.5
	31–36	4	30.8
**Number of cycles of fertility treatment used**	1–5	3	23.1
	6–10	4	30.8
	11–15	2	15.4
	16–20	2	15.4
	21–25	1	7.7
	Missing data	1	7.7
**Utilisation of at least one round of IVF**	Yes	12	92.3
	No	1	7.7
**Use of fertility treatment only in Israel**	Yes	11	84.6
	No	2	15.4
**Time using fertility treatment (years)**	< 1	1	7.7
	1–3	6	46.2
	4–10	5	38.5
	> 11	1	7.7
**Treatment outcome experienced**	Live birth	11	84.6
	Pregnant at interview	6	46.2
	Loss up to 20 weeks	6	46.2
	Loss after 20 weeks	1	7.7
**Current treatment status**	Plan to restart treatment	7	23.1
	Undecided	2	15.4
	No plan to restart treatment	4	30.8
**Reason not planning to restart treatment**	Conceived naturally	1	7.7
	Hope to conceive naturally	1	7.7
	Family complete	2	15.4

**Fig 1:**
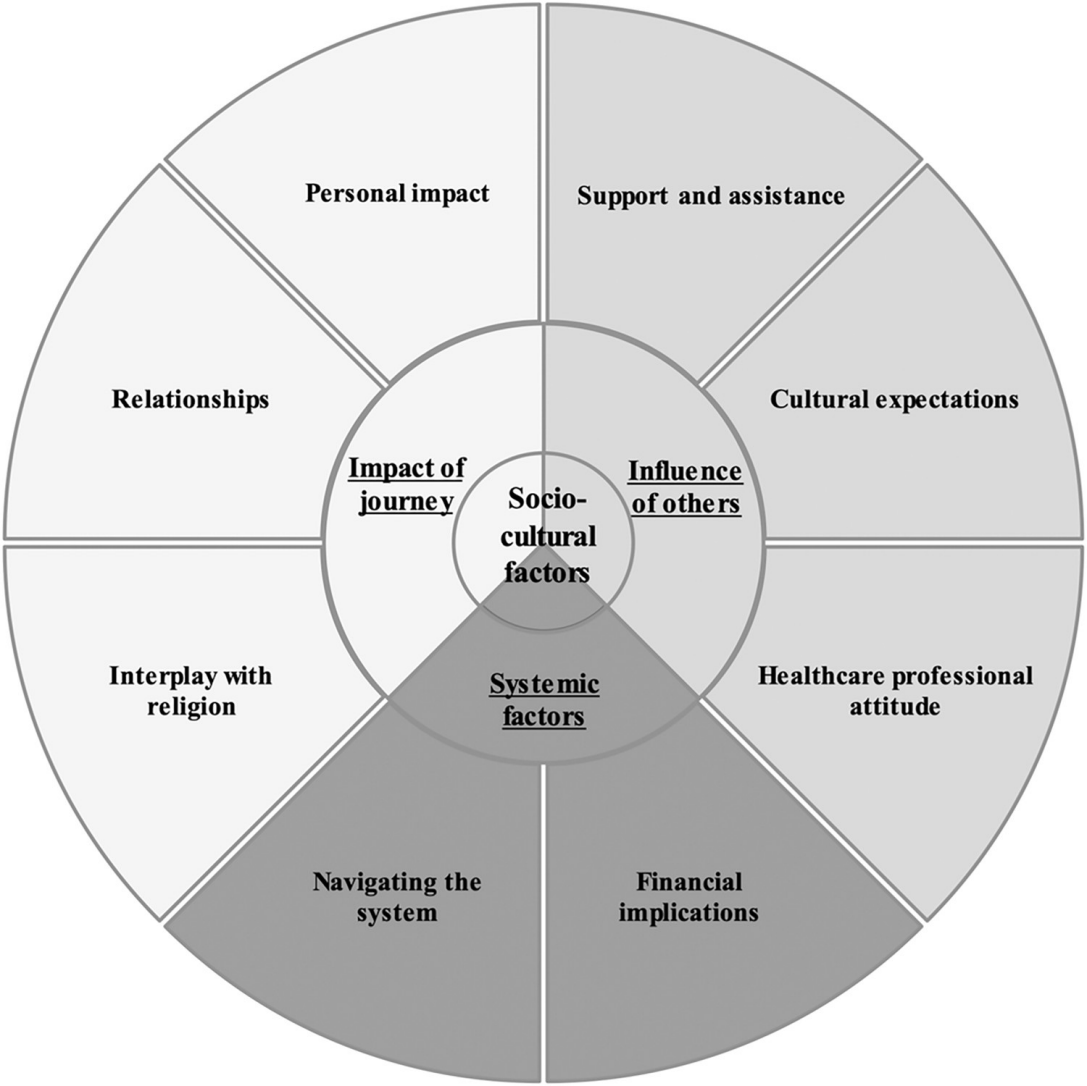
Themes and subthemes. The three key themes and their subthemes are displayed, with the underlying concept of the impact of socio-cultural factors shown in the centre.

Three key themes were developed: systemic factors, influence of others and impact of journey. These themes, alongside their subthemes, are displayed in Fig 1. Socio-cultural factors were a central concept connecting each theme.

### 1. Systemic factors

#### Financial implications

Participants “*didn’t feel a financial burden”* (P1) when commencing treatment or the need to limit the number of cycles they used due to affordability. Participants felt *“grateful”* (P13) to have received fertility treatment in Israel since finances did not determine family size.

“*… if I was living in a different country, I probably wouldn’t be able to afford [fertility treatment], let alone as many cycles as I ended up needing to conceive”* (P1)

Due to co-payments fertility treatment did still incur a minor cost, however, this was like *“pennies”* (P1) compared with other countries. Without a major financial burden, participants were able to focus on their “*actual emotions”* (P1), rather than what else could be done with the money.

“*… each failed round I wasn’t like, oh my God that’s money for my house, oh that’s my mortgage”* (P9)

Some grievances with the provision of fertility treatment were accounted to the treatment being state funded. These grievances included a perception of acceptance towards failed cycles since the patient was not paying for it and a worse attitude to patient care. The low cost of treatment made these frustrations with the Israeli healthcare system bearable.

“*Well, it would have been completely unacceptable if I was paying for this. The only reason I’ve lasted so long here is because it costs virtually nothing”* (P7)

#### Navigating the system

Many participants found the system *“bureaucratic”* (P12) struggling with the excessive *“responsibility on the patient”* (P7) and knowing “*what each doctor wanted”* (P10).

“*I have to call the hospital for this type of test, you have to call Maccabi [a healthcare service] for this type of test … it’s very decentralised. It can be hard to know what you need, and who to go to for what.”* P7

Participants also struggled with the time demands of the treatment process, often waiting “*for hours”* (P10) to be seen and spending days waiting for phone calls.

“*You’d get a blood test and you would wait for the phone to ring … God forbid you missed the phone call, and you had to try to call the office back, and it would not work”* P5

Participants were also displeased with the overall lack of *“continuity”* (P12) whereby their management plan could be changed by any of the health centre’s doctors as opposed to one designated doctor.

“… *any doctor can look at [your file] and any doctor can update what you should be doing … which was also very frustrating and very worrisome for me”* P1

Although participants found that most doctors spoke English, other healthcare professionals or administrative staff often did not. The language barrier was “*very nerve racking”* (P1). There was also a lack of resources in English with more information available in Russian and Arabic.

“*… even though I have decent Hebrew … it was definitely an added element of anxiety to walk into like a medical setting, which is really important to understand… I had to like limit myself to what I could actually express”* P12

Participants felt part of a “*mass machine”* (P9), which was “*very systematic*, *for better and for worse”* (P13). Overall, a “*desire for improvements”* (P13) is needed, for example through employing “*built in patient advocates for olim [immigrants to Israel]*” (P12) and improving access to English resources.

### 2. Influence of others

#### Healthcare professional attitude

Participants experienced a *“different bedside manner expectation”* (P12) in Israel than in their native country. The *“culture shock”* (P8) of dealing with doctors who were “*so cold*, *and so not caring”* (P8) led to participants feeling *“uncomfortable”* (P1).

“*… do you choose somebody with bedside manner who is not necessarily the best doctor, or somebody who you know is a superstar, but they’re going to make you feel awful”* P13

Since *“Anglos really like to have their hands held”* (P13), participants felt the attitude whereby healthcare professionals were unwilling to spend time answering questions or provide in-depth explanations of the woman’s circumstance was *“harmful … and frustrating”* (P13).

“*… they are just rude, impatient, they don’t explain things”* P2

Insufficient emotional sensitivity within the fertility system and a *“lack of mental health awareness*” (P8) was experienced. Despite this, some participants felt well supported in a logistical sense.

“*Israeli society is not into emotional support, they’re very logistics focussed, they will emotionally support you by helping you practically”* P5

Further comfort came from having a healthcare team who understood the participants both religiously and culturally.

“*… my doctor was completely secular, but just before he did the transfer, he said a blessing, in Hebrew … that was such a powerful experience, and I knew I wouldn’t get that anywhere else, like in the world”* P5

Through understanding Jewish rules the healthcare team offered guidance regarding the religious elements of fertility treatment.

“*… after my egg retrieval the nurse said to me ‘you’re going to bleed, but you’re not in niddah [Jewish practice whereby a woman is not permitted to touch her husband during her menstrual period nor for seven days afterwards] because it’s a clear scrape’”* P12

#### Cultural expectations

Israeli society was described as *“family focussed”* (P5) and participants explained that within religious communities “*it’s a cultural expectation”* (P5) to have children early in marriage.

“*… within our circles, obviously sort of religious people… you kind of feel the pressure if you’re not pregnant”* P11

Whilst some participants felt the heightened pressure in Israel was due to increased religiosity compared to their home country, the pressure to have children also extended into secular Israeli communities.

“*I don’t think I’d have four kids living in the UK … I think that a lot of people [in Israel] tend to have more kids … even if you’re not religious”* P3

Israel has “*much more of an open culture”* (P9) than the participants’ native countries. Whilst on the one hand this resulted in experiences of intrusive comments regarding family planning that made it “*very difficult to be in social situations”* (P5), it also meant that the expectation to have children allowed the participants’ families and friends to act more sensitively and anticipate a fertility problem and participants felt understood when accessing fertility treatment at a young age. Despite this, not everybody had “*emotional intelligence”* (P13) and participants identified a need for *“more awareness”* (P10) amongst the general public about questioning family circumstance.

#### Support and assistance

Fertility treatment is a *“difficult experience”* (P5) and participants used infertility support groups for emotional support as well as practical advice. These groups helped to *“fill in gaps”* (P13) since participants found there was “*very little emotional [support] provided”* (P10) by the healthcare system. In particular, participants felt that leaning on other migrants who had also been through the fertility treatment process in Israel was most helpful as they “*understand the culture shock with you”* (P5). Facebook groups were also a *“game changer”* (P12), providing women with a community where their experiences were normalised and reassuring them that they were “*not alone”* (P13).

“*… [leaning on] an immigrant is helpful because again, they know … what is a big deal versus what’s not a big deal, because you come from that shared immigrant background”* P5

Many participants did not utilise support offered by the healthcare system since it was in Hebrew and they were more comfortable discussing emotions using their preferred language.

“*… there was a support group that I could join, within the system. I didn’t join it, because I knew it was going to be Hebrew, and I knew that just wouldn’t do it for me … as soon as I’m emotional the words cannot come out”* P13

### 3. Impact of journey

#### Interplay with religion

Certain aspects of the Jewish religion influenced participants’ experiences of their fertility treatment journey. Niddah may have been a cause of infertility for some participants since they may have not been able to go to the mikvah (ritual bath in which a woman must immerse at the end of niddah before she may resume physical intimacy with her husband) until after they had ovulated, thereby missing their chance to conceive that month.

“*The rabbis try to make it very lenient, so the problems that you’re having with bleeding and stuff don’t hinder your ability to have a child … they’ll make leniencies to go [to the mikvah] early so you can catch your ovulation.”* P13

Some participants experienced fertility treatment to be a “*difficult time religiously”* (P5). The laws of Niddah were particularly challenging.

“*… every single month you have like a reminder that you’re kind of failing at what you want to achieve and while you’re dealing with the emotions of that you also have this kind of like physical distance from your spouse … that also challenged, therefore, my relationship with God, because it felt like even more of a punishment”* P1

Other participants took comfort from religion as it provided an explanation for their difficulties.

“… *as a religious person, you have to like see some religious meaning in life … I have to assume that there’s a reason that we’re going through this craziness”* P7

#### Relationships

Whilst participants felt fertility treatment puts *“stress on the couple”* (P11), overall they had a “*deeper love and affection”* (P4) for their partner.

“*… [participant’s husband] would be like I don’t want to do this anymore, it’s ruining our family, but I couldn’t give up … our struggles have sometimes pulled us apart a little bit, but they have also brought us together.”* P13

Engaging within communities where those around them were having lots of children was challenging. Ultimately many participants turned *“inwards”* (P10) by withdrawing from their community and socialising less.

“*I live in a very like fertile community … when you’re going through treatment, and everybody else around you is pregnant, having babies, and you can’t get there … it’s too overwhelming”* P13

Participants carefully selected who to disclose their journey to considering who would be *“amazing and supportive”* (P3). Although sharing helped participants access support, participants struggled with the need to provide updates regarding their treatment.

“*I didn’t really want anybody knowing my business because it would have felt like a monthly check in of like did it work, did it not work”* P1

#### Personal impact

The participants felt that their lives *“revolved around the treatments”* (P5) and the process forced them to put their lives *“on a hold”* (P2).

“*… my biggest frustration about the whole process … was how time consuming it is … you have a million and one tests to do. You’ve got so many doctors to run to here, there, and everywhere”* P2

The treatment process placed a significant strain on participants’ lives, even impacting on their ability to keep jobs. Multiple participants felt grateful to be in jobs that gave *“flexibility”* (P13). Despite this, there was a perception of having *“missed more work”* (P7) going through the process in Israel rather than elsewhere due to the appointment burden, long amounts of time spent in waiting rooms before being seen and overall system inefficiency.

“*I’ve actually had to make choices over my career because of fertility treatments, I’ve had to turn down dream jobs, because I knew that I couldn’t do both at the same time”* P13

Throughout the treatment process participants experienced an *“emotional toll”* (P13) and both *“physically and mentally”* (P3) did not feel like themselves. Particularly following treatment failures participants felt *“guilty”* (P6) or like a *“failure”* (P10). Participants had varying coping mechanisms throughout the process including finding *“kid free spaces”* (P5), changing their *“attitude to stress”* (P2) and making sure they kept *“having fun”* (P3). Overall, most participants had *“trust in the health system”* (P6), with one participant stating Israel is “*the best place in the world”* (P13) for fertility treatment.

## Discussion

This study aimed to explore the experiences of English-speaking women who migrated to Israel and subsequently used largely state funded fertility treatment. The main findings were often influenced by socio-cultural factors. Such factors included the influence of others, systemic factors in the Israeli healthcare system and the overall impact of the journey on these women’s lives.

Since fertility treatment in Israel is largely state funded through compulsory universal national health insurance, women can continue treatment until they conceive without having to consider affordability [14]. In a healthcare system where women are required to pay for fertility treatment, a common reason for treatment discontinuation was financial problems [33]. However, in Israel, finances do not determine family size in the same way. The lack of a financial barrier to treatment in Israel has led to a culture of perseverance with fertility treatment whereby women undergo high numbers of treatment cycles [14]. This was reflected by the study’s sample with the majority of women utilising more than 5 treatment cycles.

The issue of bureaucracy within the Israeli healthcare system was repeatedly highlighted. Participants experienced the system to be confusing and there was a perception of acceptance towards failed cycles and a poor attitude to patient care. Many of these issues were accounted to the treatment being state funded resulting in IVF units being overrun [34]. Participants believed the treatment process would have imposed less of a burden on their lives in countries such as the US where fertility treatment is provided through private healthcare [35]. Despite the negative aspects of the treatment process, once participants were in the rhythm of treatment it was systematic and they appreciated the low cost incurred.

Globally research into the reproductive health of migrants focusses on those who have migrated due to wider issues, mostly humanitarian emergencies. This study’s population of English-speaking migrants to Israel did not face such challenges. Their main challenge resulted from contending with the ‘cultural imperative’ to have children at an already emotionally trying time [5].

In Israel all Jewish communities, including secular women, have higher fertility rates than their counterpart communities with similar levels of religiosity in the US [9]. A study found that the difficulties experienced by fertility patients in Israel are similar for women with no children or with one child and only improved for those women who had two children. This suggests that in Israel having just one child still does not relieve the stress of infertility [36]. Interestingly, data also show that fertility rate increases amongst migrants to Israel demonstrating the impact of the pronatalist culture [9].

Within a society whereby having children is expected infertile couples do not have the option to hide their struggle [37]. In alignment with this, participants experienced intrusive questions regarding their family planning that they did not believe would have been asked in their country of origin. An Israel-based study identified questions and social pressure about childbearing as being amongst the most prominent difficulties experienced by women during fertility treatment [36]. Participants withdrew from their community and spent less time with friends and family as socialising with those who were successfully having children was too challenging. This parallels literature that identified that for women who are unable to have children, spending time within their communities can emphasise the pain of being unable to conceive [38]. Furthermore, a study based in Israel identified social withdrawal as a coping mechanism during infertility treatment and one of the major correlates of distress [39]. Despite these struggles many participants received support from family and friends and identified utilisation of fertility treatment in Israel as normalised. This finding is unsurprising considering Israel has the highest rate of IVF per capita worldwide [40].

Whilst navigating fertility treatment in Israel participants relied on support from fellow migrants who were able to provide the most effective assistance as they best understood which aspects of the journey would present a culture shock. Other support came through online Facebook groups or worldwide organisations, such as UK based organisation Chana [41]. A study into stress in fertility treatment patients in Denmark found high levels of social support and disclosure of infertility to close relations reduced the stress associated with infertility [42]. These support networks were required since there was a lack of emotional support provided by the healthcare system. In a study into the perceptions of patient-centred care in Israeli IVF units, patients gave low scores for provision of emotional support [43]. Any available support through the healthcare system was in Hebrew, but this study found, in alignment with existing research, women are more comfortable discussing emotional topics in their preferred language [44]. An existing study regarding perseverance with fertility treatment despite failures advised centres providing treatment to improve provision of psychological support [14]. This is in contrast to countries such as the UK whereby the Human Fertilisation and Embryology Authority only licences clinics to provide fertility treatment if they also offer access to a counsellor [45].

Although all participants had at least a basic Hebrew language ability they were concerned about misunderstanding information in a healthcare setting. Accessing resources in English was challenging with more resources translated to Arabic or Russian. This is likely because 20% of the Israeli population are Arabs and most migrants to Israel since the early 1990s came from countries that were previously part of the Soviet Union [46, 47]. The Israeli Central Bureau of Statistics identified 75% of immigrants to Israel in 2023 being from Russia or Ukraine [25]. No literature investigating English-speaking migrants’ experiences of navigating the Israeli healthcare system was found for comparison. Despite this, the participants’ experiences are supported by an internet search which showed Israel’s English Ministry of Health website is much more limited compared with the Hebrew website. When searching ‘fertility’ in Israel’s English Ministry of Health website 218 results appear compared with 1300 results when the Hebrew website is searched [48].

The attitude of impatience, rudeness and coldness within the Israeli healthcare system was different to the attitude of healthcare professionals that participants were used to in their native country. This poor bedside manner negatively impacted upon the participants’ experiences in alignment with a study that found decreased communication between nurses and patients leads to increased feelings of isolation [49].

Regardless of the religion in question, or level of religiosity, undergoing fertility treatment often presents unique religious and spiritual needs [50]. As all respondents were Jewish, undergoing fertility treatment within a system whereby many of the healthcare professionals understand the Jewish religion’s rules and cultures is unique to Israel and this improved the experience of participants. Existing literature explains that understanding of religious beliefs allows more culturally competent care [51]. Often participants used religion as a coping mechanism aligning with the finding that connectedness with God can reduce psychological distress [52]. Infertility also challenged faith in God. This concept is supported by an existing study which found religion gave women a positive outlet throughout their infertility journey. Despite this, the study identified that religious observance reduced with the number of infertile years experienced [49]. Overall, it is important to address the varying concerns that arise due to the interplay of religion and fertility treatment [53].

### Strengths and limitations

This study began to address the gap in knowledge regarding the experiences of English-speaking women who migrate to Israel and use Israeli healthcare services. Whilst member validation was not possible due to time and resource constraints, coding of transcripts by two researchers and discussion of themes within the research team improved validity [54]. Data saturation was reached within the study’s population since no new codes or themes were identified by the 13th interview [55]. Nevertheless, due to a lack of variation within the population, saturation was not reached for the topic as a whole. Within the population there were varying levels of Hebrew language ability, country of origin and age of migration to Israel. However, all 13 participants were Jewish, of whom 10 were religious. Level of religiosity was likely to have influenced treatment experience. Furthermore, all participants had either had a live birth or were pregnant through fertility treatment at the time of interview which may have positively biased their retrospective accounts of their experience with fertility treatment. Recruitment of a diverse sample with women of any religion and any treatment outcome was attempted through advertisement in various Israel specific Facebook groups. Despite this, recruitment through Facebook prevented inclusion of ultra-Orthodox (Haredi) women as they tend to have limited internet usage. Furthermore, interviewing remotely allowed inclusion of women living across Israel. Since participants were required to have migrated to Israel, the lack of religious variation was likely a reflection of participants migrating under Israel’s Law of Return. This law entitles Jews to migrate to Israel [23]. Further research should target women from specific ethnic or religious groups to identify experiences of women from a wider demographic, including Arabs, Druze and other groups. Women who had not been successful with fertility treatment were unlikely to participate because talking about their experience would have been emotionally demanding. Since participants were required to speak English, they may have had different experiences of fertility treatment than migrants who speak other languages. Maintaining a reflexive approach and collaborating with a local researcher helped to mitigate the influence of cultural differences between the primary researcher (female, British, Jewish, undergraduate medical student) and the participants on findings.

### Recommendations

Fertility treatment centres, in addition to organisations designed to support migrants in Israel such as Nefesh B’Nefesh, are encouraged to increase provision of English language resources [56]. Future research is recommended into identifying how best to support women who migrate to Israel in terms of navigation of the pronatalist society as well as the healthcare system itself. This research may be most effective if targeted at specific subsets of migrants, for example by level of religiosity or country of origin as they are likely to have different experiences and needs. Research into how the attitude of healthcare professionals impacts on the experience of fertility treatment and what can be done to improve these patient healthcare professional interactions is also worthwhile.

## Conclusion

This study identifies the experiences of English-speaking women who migrate to Israel and access state funded fertility treatment. The findings begin to address the gap in the literature regarding the experiences of such women. Although all the participants had at least a basic Hebrew language ability and migrated to Israel optionally, they still faced challenges navigating the Israeli healthcare system as migrants. Interacting with healthcare professionals whose attitudes are unfamiliar and contending with a pronatalist society also negatively impacted upon the experiences of these women. Their experiences are improved by the minimal financial burden associated with treatment and the understanding of healthcare professionals regarding their religious beliefs. Barriers that these women face must be better anticipated. Improved provision of resources in English is essential and further research is needed into how women can best be supported whilst navigating Israel’s pronatalist society.

## Supporting information

S1 Checklist Inclusivity in global research checklist. (DOCX)

S1 File Topic guide. (DOCX)
