# Supplementary material for: Exploring the experiences of English-speaking women who have moved to Israel and subsequently used Israeli fertility treatment services: A qualitative study
Source: PLoS One. 2024 Aug 28;19(8):e0309265. doi: 10.1371/journal.pone.0309265 (PMC11356427; doi:10.1371/journal.pone.0309265)
Supplement: S1 File — (DOCX) [file pone.0309265.s002.docx]

**Topic Guide**

| Topic | Questions and Prompts |
| --- | --- |
| Establishing participant circumstance | Why did you decide to use fertility treatment?   - What was your experience with attempting to become pregnant prior to this?   What was your experience with fertility treatment before seeking it for yourself?   - What did you know about the process before you started receiving treatment? And how has this changed? |
| Disclosure | Who did you tell that you were attempting to get pregnant using fertility treatment?   - What factors influenced who you shared this with?   How did sharing this information with these individuals affect your experience?   - In what ways was this helpful? - In what ways was sharing information about your experience unhelpful? - How did telling these individuals about your treatment affect your relationships with them?   Did your experience of sharing information about the treatment process change over time? |
| Barriers and facilitators | How did you deal with the treatment process?   - What factors improved your experience? - Did this change over time?   What difficulties did you experience during the treatment process?   - What helped you overcome these difficulties?   Did you access support services?   - What services were they and what was your experience with them?   If at all, how has the treatment process affected your career?   - Have you had to take time off work? If so, what was your experience of this?   How have your religious or cultural beliefs affected your experience of the fertility treatment process? |
| Experience with healthcare services and professionals | How did you find the process of accessing treatment?   - What worked well when it came to accessing treatment? - What didn’t work well when it came to accessing treatment?   What was your experience with the healthcare professionals?   - What emotional support was offered by the healthcare staff? - Is there anything more you think the healthcare professionals could have done to help you through the process?   Were there any troubles with regard to language or culture barriers?  How would your treatment experience have been affected if you had to pay?   - In what way did treatment being free impact your decision to commence treatment? |
| Impact on life | How did the treatment influence your relationships?   - Including family, friends, partner   Did you find a difference in how individuals living in Israel vs those not responded to your treatment?   - If so, how? - If so, why do you think this is the case?   How did the treatment process affect your lifestyle?   - Do you feel undergoing the process in Israel vs [original country of origin] influenced this and if so, how?   How did you feel undergoing the treatment in a country that you haven’t lived in for your whole life?   - How did this affect your experience? - Were there any particular aspects of the journey that you feel were specific to undergoing treatment in Israel? |
| Outcome of the process | If they haven’t had a child yet   - Have they stopped treatment – if so why and how do you feel about this? - Are they carrying on with treatment – if so why and how do you feel about this?   Has your experience changed over the duration of the treatment process?   - If yes, how? |

**Final questions**

- What do you think could be done for women such as yourself, who haven’t lived in Israel all their lives, to improve the overall experience of the fertility treatment process?
- What is your overall reflection on your experience of using Israeli fertility treatment services?
- Is there anything else that you think is important for me to know about the experiences of women who have emigrated to Israel and utilised fertility treatment services?
